# Methazolamide improves neurological behavior by inhibition of neuron apoptosis in subarachnoid hemorrhage mice

**DOI:** 10.1038/srep35055

**Published:** 2016-10-12

**Authors:** Mingchang Li, Wei Wang, Haojian Mai, Xinmu Zhang, Jian Wang, Yufeng Gao, Yuefei Wang, Gang Deng, Ling Gao, Shuanhu Zhou, Qianxue Chen, Xin Wang

**Affiliations:** 1 Department of Neurosurgery, Renmin Hospital of Wuhan University, Wuhan, Hubei, 430060, P. R. China; 2 Department of Neurosurgery, Brigham and Women’s Hospital, Harvard Medical School, Boston, MA, 02115, USA; 3 Department of Neurosurgery, Baoan District Hospital, Shenzhen, Guangdong, 518100, P. R. China; 4 Department of Anesthesiology and Critical Care Medicine, The Johns Hopkins University, School of Medicine, Baltimore, MD 21205, USA; 5 Department of Endocrinology, Renmin Hospital of Wuhan University, Wuhan, Hubei, 430060, P. R. China; 6 Department of Orthopedic Surgery, Brigham and Women’s Hospital, Harvard Medical School, Boston, MA, 02115, USA

## Abstract

Subarachnoid hemorrhage (SAH) results in significant nerve dysfunction, such as hemiplegia, mood disorders, cognitive and memory impairment. Currently, no clear measures can reduce brain nerve damage. The study of brain nerve protection after SAH is of great significance. We aim to evaluate the protective effects and the possible mechanism of methazolamide in C57BL/6J SAH animal model *in vivo* and in blood-induced primary cortical neuron (PCNs) cellular model of SAH *in vitro*. We demonstrate that methazolamide accelerates the recovery of neurological damage, effectively relieves cerebral edema, and improves cognitive function in SAH mice as well as offers neuroprotection in blood- or hemoglobin-treated PCNs and partially restores normal neuronal morphology. In addition, western blot analyses show obviously decreased expression of active caspase-3 in methazolamide-treated SAH mice comparing with vehicle-treated SAH animals. Furthermore, methazolamide effectively inhibits ROS production in PCNs induced by blood exposure or hemoglobin insult. However, methazolamide has no protective effects in morality, fluctuation of cerebral blood flow, SAH grade, and cerebral vasospasm of SAH mice. Given methazolamide, a potent carbonic anhydrase inhibitor, can penetrate the blood–brain barrier and has been used in clinic in the treatment of ocular conditions, it provides potential as a novel therapy for SAH.

Subarachnoid hemorrhage (SAH) is a hemorrhagic stroke caused by a variety of factors, more than 90% of which are aneurysm and arteriovenous malformation^1^. With high mortality and morbidity of SAH, it usually causes poor outcome and occupies about 5% in all cerebrovascular accident. It has been reported that about 12% of patients died before treatment, 33% suffering from death within 48 hours, and the mortality rate is as high as 50% within 30 days^2^. If not given the right treatment in time, the patients survived for the first bleeding may occur with bleeding again within 3 weeks, the mortality of which is as high as 80%^3^. About 30% of the survivors suffered from varying degrees of nerve dysfunction, such as hemiplegia, mood disorders, cognitive and learning and memory impairment. The latest preclinical and clinical study show that SAH resulted in a series of complex pathophysiological process, including the intracranial pressure increasing, cerebral blood flow and cerebral perfusion pressure reducing, blood-brain barrier (BBB) damage, brain edema, acute cerebral vasospasm, microvascular dysfunction and neuron apoptosis mechanism^4^. The above process can secondary cause glutamic acid poisoning, calcium overload and free radicals accumulation, mitochondrial dysfunction, glial cells overactive autophagy activity and immune inflammatory reaction. Although there are many studies on this pathological mechanism, no clear measures can reduce the brain damage. Therefore, looking for effective measures is still an important task for our neural science researchers.

Methazolamide (Neptazane) has been indicated in the treatment of ocular conditions^5^. It is a potent carbonic anhydrase inhibitor that can penetrate the BBB, which is proved to have the effect of neuroprotection. We previously reported that methazolamide, not only inhibits the release of mitochondrial cytochrome C and reduces the activation of caspase 9 and caspase 3 in the treatment of chronic neurological disorder Huntington’s diseases^6^, but also reduces the infarction area and improves neurological function score in mouse model of acute ischemic stroke^7^, while methazolamide plays a role in cerebral cortex cells and inhibits the release of cytochrome C, further inhibits cell apoptosis to achieve the protection of cerebral cortex neurons^7^. However, to our knowledge, no study has investigated if methazolamide eventually leads to an improvement in neurological outcomes after experimental SAH.

In this study, we used intravascular puncture SAH model in mice and observe the effect of methazolamide on the nerve function and apoptosis of neurons after SAH in mice. The aim of this study was to examine the protective effects and the possible mechanism of methazolamide in SAH, providing a new method for the treatment of SAH.

## Methods

The methods were carried out in accordance with the approved guidelines.

### Experimental animals

All the protocols used in this study were approved by the Institutional Animal Care and Use Committee at Wuhan University. One hundred and fifteen adult male C57BL/6J mice (provided by the laboratory animal center of Wuhan University Medical College) weighting between 25 and 30 g were randomly assigned to the following 3 groups: sham, SAH + vehicle, SAH + methazolamide (20 mg/kg). Methazolamide or vehicle was administered 30 min after surgery, and outcomes were assessed in the specific time.

### Subarachnoid hemorrhage modeling

The endovascular perforation was produced as previously described with modifications^8^. Anesthesia was induced with sodium pentobarbital (80 mg/kg). After anesthesia of intraperitoneal injection in mice, a laser Doppler flowmetry probe (Moor Instruments Inc., Wilmington, DE) was adhered at the left temporal bone to monitor the blood flow of the left hemispherea. The left common carotid artery (CCA), internal carotid artery (ICA) and external carotid artery (ECA) were exposed and the external carotid artery (ECA) was fashioned into a 3-mm stump. A 6-0 monofilament nylon suture was advanced into the internal carotid artery (ICA) from the ECA stump until resistance was felt, about 1.0 cm from Common carotid artery bifurcation have resistance to insert again after 2 mm pricking blood vessels and then the suture was immediately withdrawn to induce subarachnoid hemorrhage. The cerebral blood flow decreased obviously on doppler blood flow meter monitoring. In the sham group, the procedure is similar, but not pricking blood vessels, blood flow is also no obvious change. We recorded the blood flow changes before and after the process of blood vessel puncture in all animals. The animals were returned to their cages after operation and had free access to food and water.

### Methazolamide administration

Methazolamide was purchased from Sigma-Aldrich, St. Louis, MO. The SAH + methazolamide group received 20 mg/kg of methazolamide intraperitoneally 30 min after SAH induction, administered once every 12 h for no more than 7 days. The drug was dissolved in phosphate-buffered saline (PBS 0.1 mol/L, pH 7.4) that containing 3% DMSO. The SAH + vehicle group received the same volume of PBS intraperitoneally 30 min after SAH induction.

### SAH grading

In order to excluded the bias results caused by the amount of bleeding in each group, each group of the experiment mice were sacrificed after 24 h, SAH grade was determined by the high-resolution images of the blood clots in the basal cisterns, as previously described by Sugawara^9^. According to the total score, it can be rated as the following three groups: 0–7 points: mild SAH, 8–12 points: moderate SAH, 13 to 18 points: severe SAH.

### Neurological Score

We used the Garcia test to evaluate animal neurological function at 24 h, 48 h, 72 h after SAH, which allowed a total score of 18 and higher scores indicated greater function^10^. Two investigators involved in the experiment with single blind method respectively to score and record of experimental mice, then the average of the two groups was used as the final score.

### Brain water content (BWC)

Animals were sacrificed at 24 h after SAH and the brains were quickly removed (n = 9). The brains were separated into left and right hemispheres, brainstem, and cerebellum. The specimens were quickly weighed (wet weight), then placed inside the oven at 100 °C for 24 h, and reweighed (dry weight). Water content in formula: (wet weight - dry weight)/wet weight × 100%.

### Open-field test

We used the open-field test to evaluate cognitive function at 3d, 7d, 14d after experiment^11^. Briefly, a 72 cm × 72 cm × 60 cm square box without cover, the center of a side length of 54 cm square for the central region and the remaining part for the edge regions. The box was placed in a well-lit place, mounted directly above a video camera, the mice were placed in the center of the open field, allowing freedom of movement. Recording the behavior of mice 5 min in the box, viewer software was used to analyze the movement of each mouse by total distance and the time to the central grid.

### The detection of cerebral vasospasm

India ink/gelatin cerebral vascular perfusion method was used to detect the average diameter of the anterior cerebral artery, middle cerebral artery and internal carotid artery on the bleeding side, as previously described^12^. Briefly, each group of mice were anesthetized by intraperitoneal (n = 8), cut the right atrium, perfuse the heart with normal saline until clear liquid flowed out from the right atrium. After replaced with 4% paraformaldehyde (PFA) by 30 ml, then replaced with 50 °C 20% India ink/gelatin solution for 30 seconds, pressure control for 80–100 mm Hg. Whole mice placed in the refrigerator at 4 °C for 24 h, decapitated and took skull base pictures. Image J software was used to calculate the average artery diameter on the bleeding side.

### Histopathological assessment

Each group of brain tissue paraffin sections were deparaffinized, placed in citrate antibody repair solution, heated to 100 °C for 8 min. Adding 3% hydrogen peroxide at room temperature for 10 min, then added antibody working solution (active-caspase-3 dilution of 1: 100), sham group was instead of primary antibody with PBS, 4 °C overnight. The biotin-labeled secondary antibody working solution was added dropwise, incubated at room temperature for 1–2 h. Prepared DAB solution was added dropwise, and immediately placed Mayer’s hematoxylin dyebath for 5–10 min. Fengpian and microscopy. The positive rate according to a formula: (positive cells/ field of all cells)× 100%. Fluoro-Jade B (FJB) staining and Terminal Deoxynucleotidyl Transferase dUTP Nick End Labeling (TUNEL) methods were also used to detect the corresponding positive cells and microscopic count.

### Western blot

Mouse brain samples ofindicated groups were lysed in RIPA buffer with protease inhibitors^7^. Cleaved Caspase-3 (Asp175) antibody was purchased from Cell Signaling Technology^13^, and antibody to GAPDH from Sigma.

### Primary cortical neuron cultures and induction of cell death

Cerebral cortex of mouse embryos at day 15 (E15) were freed from meninges and separated from olfactory bulb and hippocampus^14^. Trypsinized cells were suspended in medium [neurobasal medium with 2% (vol/vol) B27 supplement, 2 mM glutamine, 100 units/ml penicillin, and streptomycin] and seeded at a density of 2 × 10^4^ per cm2 on polylysin-coated 96-well plates or dishes. Cells were used for experiments on day seven of culture. 1, 2.5, 5 μl fresh blood collected using retro-orbital vein method in mice are added to the 100 μl medium. Primary cortical neurons (PCNs) were exposed to indicated proper amount of blood or indicated concentration of hemoglobin, either with or without methazolamide. Forty-eight hours after treatment with blood, PCNs were either submitted for microtubule-associated protein-2 (MAP2) immunostaining and counting or for Reactive Oxygen Species (ROS) ELISA assay.

### Detection of intracellular ROS

The ROS ELISA assay was performed as described according to the manufacturer’s instructions (MyBioSource, Inc). Briefly, PCNs were grown in 96-well plates and exposed to indicated amount of blood or hemoglobin, either with or without methazolamide for 18 h. Mouse ROS monoclonal antibody was pre-coated in a ELISA plate. PCNs were trypsinized and centrifuged for 10 min at 3000 rpm. The supernatants were added into ELISA plate wells and incubated for 90 min at 37 °C incubator, the detecting biotin labeling polyclonal antibody was added into ELISA plate and hatched for 60 min at 37 °C. Then the avidin-peroxidase conjugates were added into ELISA plate and incubated for 30 min at 37 °C. TMB substrates for coloring were added into ELISA plate for 30 min. Absorbance of PCNs samples at 450 nm was measured in an ELISA reader and interacellular ROS (IU/ml) was obtained.

### Immunocytochemistry

PCNs were exposed to blood or hemoglobin with or without methazolamide. Cells were fixed in 4% paraformaldehyde for 15 min. After washing in PBS and blocking in 5% normal goat serum/0.3% Triton X-100 in PBS for 60 min, cells were incubated with anti-MAP2 antibodies (1:100) (Cell Signaling Technology) and then FITC-conjugated secondary antibodies in 1% BSA/0.3% Triton X-100 in PBS and then FITC-conjugated secondary antibodies. The number of PCNs per well were summed from the fields counted and expressed as a percent of those present in control wells that not exposed to blood or hemoglobin. Digital images were taken with a fluorescence microscope.

### Statistical analysis

Data were analyzed using the commercially available software SPSS 16.0. Statistical significance was verified by one-way analysis of variance (ANOVA), followed by Tukey’s test for multiple comparisons as appropriate. Differences in BWC among groups were compared using Fisher’s test. Significant ANOVA interactions were further explored using the conservative Scheffe post-hoc test. Difference in mortality was tested using Fisher’s exact test, with *P *< 0.05 considered statistically significant. Drug analysis, including IC_50_ and maximum protection, were performed by GraphPad Prism program.

## Results

### Methazolamide has no protective effects in morality, fluctuation of cerebral blood flow, and SAH grade of SAH mice as well as the cerebral vasospasm in SAH mice

There are three main established animal models of SAH: endovascular puncture, blood injection of cisterna magna and cross forebay^2^. Blood injection of cisterna magna and cross forebay model can control the volume of the blood in subarachnoid space, but they can not simulate the physiological effects of SAH. Intravascular puncture model simulates well the role of blood flow in a direct impact on the brain tissue, similar to the clinical scenario caused by the SAH. Therefore it makes our study clinically relevant. Since the clinical severity of the SAH patient due to the amount of bleeding, thus division has been widely used in the clinical judgment of the patient’s condition based on this principle^15^. In current study, we used endovascular puncture SAH model in mice to investigate the effect of methazolamide. The overall mortality rate of SAH was 25.3%, similar to the report in the literature^16^. No deaths in the sham group, in SAH + vehicle group mortality was 26.1%, in SAH + methazolamide group mortality was 24.4%. Mortality was not significantly different between SAH + vehicle group and SAH + methazolamide group (p > 0.05). In other words, methazolamide has no protective effects in morality of mouse model of SAH.

**Figure 1:**
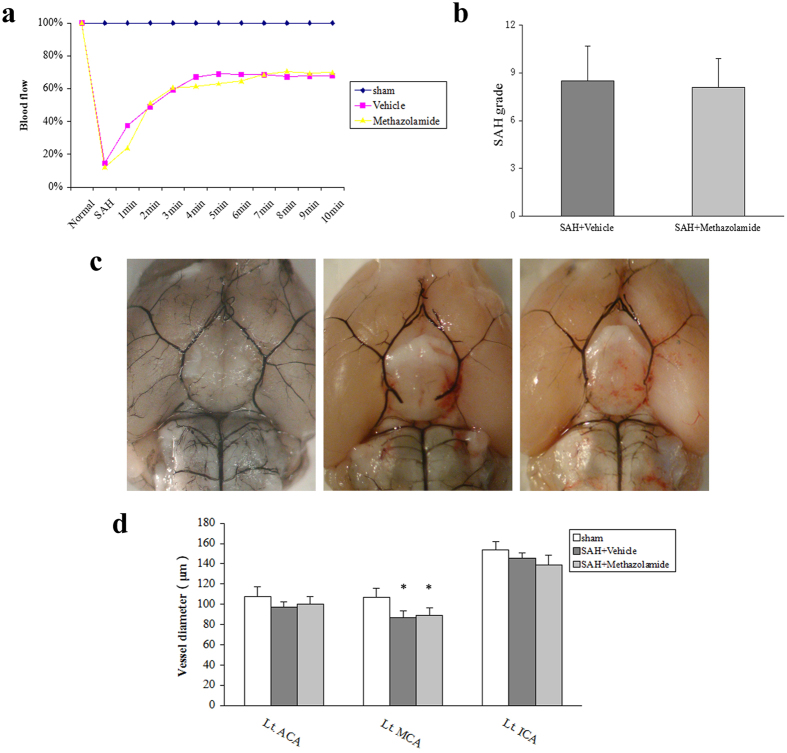
Comprasion of cerebral blood flow, bleeding grade, in each group. (**a**) The changes of cerebral blood flow. We recorded the blood flow changes before and after the process of blood vessel puncture in animal model (*P *> 0.05). No significant fluctuation was found in cerebral blood flow in the sham group. (**b**) Bleeding grade in each group. No bleeding in sham group. The average SAH grade has no significant difference (*P* > 0.05) among the two groups. (**c**) Representive image of reperfusion of SAH brain. a. the sham group. b. the SAH + vehicle group. c. the SAH + methazolamide group. (**d**) Comparsion of vessel diameter in groups. Compared with the sham group of the middle cerebral artery, the diameter of SAH + vehicle group and SAH + methazolamide group was significantly reduced (*P* < 0.05). However, the SAH + vehicle group had no significant difference (*P* > 0.05) compared with SAH + methazolamide group on vessel diameter. N = 8 mice for each group.

In order to compare the severity of SAH in each group, we recorded the blood flow changes before and after the process of blood vessel puncture in animal model. We found no significant fluctuations in cerebral blood flow in the sham group (P > 0.05, Fig. 1a). However, in the SAH + vehicle group and SAH + methazolamide group after retracement of suture, cerebral blood flow reduced to 10% of the foundation within 20 seconds, then gradually recovered to 70% after 10 minutes. In each recording time, cerebral blood flow was no significant difference between the SAH + vehicle group and SAH + methazolamide group.

The clinical severity of the SAH patient due to the amount of bleeding, thus division has been widely used in the clinical judgment of the patient’s condition based on this principle^17^. Therefore, we scored the bleeding of SAH after 24 h. The results showed there is no bleeding in sham group. The average SAH grade was 8.50 ± 2.17 in the SAH + vehicle group, 8.09 ± 1.81 in the SAH + methazolamide group. There was no significant difference in the SAH grades among these two groups (*P *> 0.05, Fig. 1b). In summary, methazolamide has no protective effects on the fluctuation of cerebral blood flow and SAH grade. Thus we excluded the bias results caused by the amount of bleeding in each group.

Each group showed no significant difference on the diameter of the anterior cerebral artery and internal carotid artery. Compared with the sham group of the middle cerebral artery, the diameter of SAH + vehicle group and SAH + methazolamide group was significantly reduced (*P *< 0.05). However, the SAH + vehicle group had no significant difference (*P *> 0.05) compared with SAH + methazolamide group on vessel diameter (Fig. 1d).

### Methazolamide accelerates the recovery of neurological damage, effectively relieves cerebral edema and improves cognitive function of animal after SAH

**Figure 2:**
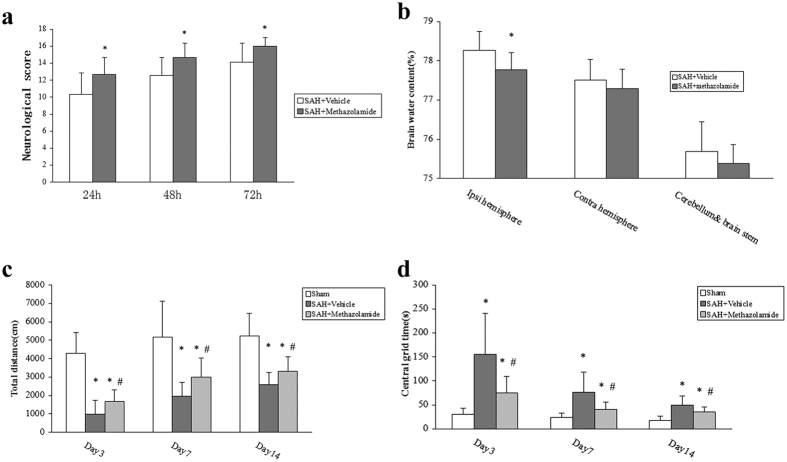
Neurological score, brain water content, movement distance in each group. (**a**) Neurological score among groups. Garcia test was used to evaluate animalneurological function at 24, 48, and 72 h after SAH, there were significant difference among 3 groups at the same time point (*P < 0.05 vs Vehicle group). (**b**) Brain water content among groups. BWC in the left hemisphere (perforation side) was significantly reduced in the SAH + methazolamide group compared to the SAH + vehicle group at 24 h post-SAH (*P < 0.05). (**c**) The total movement distance in the SAH + vehicle group and SAH + methazolamide group, sham group showed a significant difference (*P < 0.01) at the day 3, 7, and 14. But SAH  + methazolamide group improved significantly (^#^*P *< 0.05) than the SAH + vehicle group. (**d**) The central grid time in the SAH + vehicle group and SAH + methazolamide group, sham group showed a significant difference (*P < 0.01) at the day 3, 7, and 14. But SAH +  methazolamide group improved significantly (^#^*P* < 0.05) than the SAH + vehicle group. N = 8 mice for each group (**a,c,d**) and n = 9 mice for each group (**b**).

We used neurological score to evaluate the nerve damage and found that the neurological score was statistically significant (*P *< 0.05, Fig. 2) between each group at 24 h, 48 h, 72 h postoperation. Moreover, the score was lowest at 24 h after surgery in each group, which was mainly attributed to the role of surgery and anesthesia. Wheareas the score of each group gradually recovered at 48 h and significantly restored at 72 h after SAH. Compared to the SAH + vehicle group and SAH + methazolamide group, sham group at all time points was significant difference (*P *< 0.01). However, SAH + methazolamide group significantly improved neurological score than the SAH + vehicle group (*P *< 0.05). These results showed that the recovery process of the SAH + vehicle group was significantly lags behind the sham group, while SAH + methazolamide group recovered quickly than the untreated group, which suggest that methazolamide can accelerate the recovery of neurological damage in SAH mice.

There was no significant difference in BWC in the right hemisphere, cerebellum, and brainstem among the groups. BWC in the left hemisphere (perforation side) was significantly reduced in the SAH + methazolamide group compared to the SAH + vehicle group at 24 h post-SAH (*P *< 0.05, Fig. 2). Our results showed that SAH + methazolamide group significantly reduced brain tissue water content than SAH + vehicle group 24 h after SAH.

Total movement distance: compared with the SAH + vehicle group and SAH + methazolamide group, sham group showed a significant difference (*P* < 0.01) at the day 3, 7, and 14. But movement distance of SAH + methazolamide group was farther (*P *< 0.05) than in the SAH + vehicle group. Central grid time: sham group showed a significant difference (*P *< 0.01, Fig. 2c,d) compared with the SAH + vehicle group and SAH + methazolamide group at the day 3, 7, and 14. The time of SAH + methazolamide group was shorter (*P* < 0.05) than the SAH + vehicle group. The results suggested that cognitive function and excitability of animals in each group was minimum at the first three days after surgery, then gradually improved, but the recovery process of the SAH + vehicle group was significantly lags behind sham group, and SAH + methazolamide group improved quickly than the SAH + vehicle group.

### The protective effects of methazolamide on the neuron apoptosis after SAH

**Figure 3:**
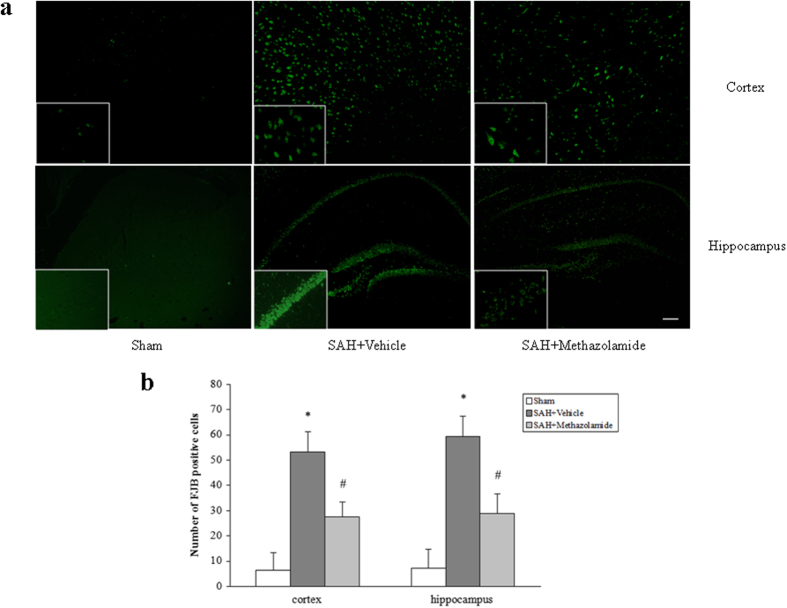
FJB staining of cortex and hippocampus of bleeding side. (**a**) Representative images of FJB-stained brain slices from cortex (top) and hippocampus (bottom) at 24 h post-SAH. Scale bar = 50 μm. (**b**) The positive cell rate of the SAH + vehicle group and SAH + methazolamide group were increased (*P < 0.01) compared with sham group, while the positive rate of SAH + methazolamide group was lower than the SAH + vehicle group (^#^*P *< 0.05). N = 5 mice for each group.

**Figure 4:**
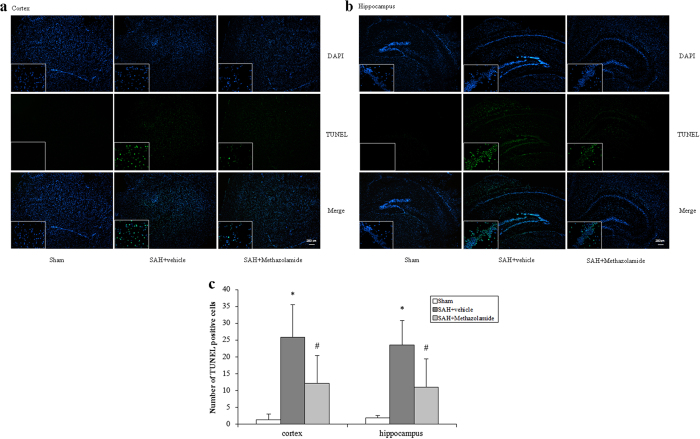
TUNEL staining of cortex and hippocampus of bleeding side. (**a**) Representative images of TUNEL staining of cortex. (**b**) Representative images of TUNEL staining of hippocampus. Scale bar = 200 μm. (**c**) The positive rate of the SAH + vehicle group and SAH + methazolamide group were increased (*P < 0.01) compared with sham group, while the positive rate of SAH + methazolamide group was lower than the SAH + vehicle group (^#^*P* < 0.05). N = 5 mice for each group.

From FJB staining and TUNEL staining, either in cortex or hippocampus of bleeding side, the positive cell rate of the SAH + vehicle group and SAH + methazolamide group were increased (*P *< 0.01) compared with sham group, while the positive rate of SAH + methazolamide group was lower than the SAH + vehicle group (*P *< 0.05, Figs 3 and 4).

### Methazolamide protects the neuron apoptosis by inhibiting the expression of active caspase-3

Recent study showed that methazolamide primarily inhibited the release of cytochrome C, further reduced mitochondrial membrane depolarization and caspase-1 and caspase-3 activation as well as inhibited the release of mitochondrial factor, finally inhibited the apoptosis so as to achieve the protective effect of cerebral cortical neurons^7^. To explore specific molecular mechanisms of inhibition of neuronal apoptosis by methazolamide in SAH, we detected the expression of active caspase-3 of the cerebral cortex and hippocampus neurons, which was a significant apoptosis-related protein.

**Figure 5:**
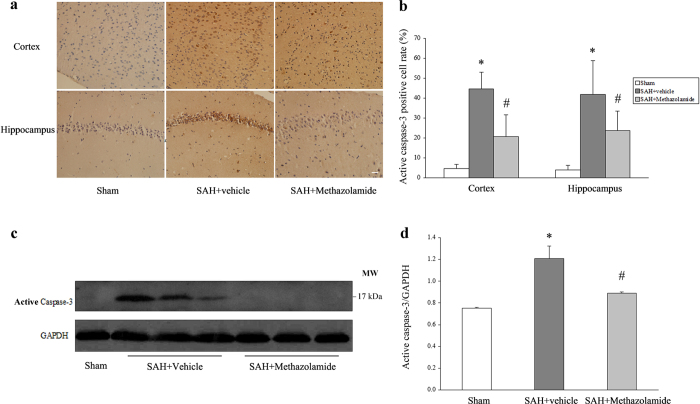
Immunohistochemistry and western blotting of active caspase-3. (**a**) Representative images of immunohistochemistry staining of cortex and hippocampus. Scale bar = 20 μm. (**b**) Bar graph shows a low positive rate of the sham group, the positive rate of the SAH + vehicle group were increased (*P < 0.01) compared with sham group, while the positive rate of SAH + methazolamide group was lower than the SAH + vehicle group (^#^*P *< 0.05). (**c**) Representative images of Western blot between different groups. (**d**) Western blot quantification showed that the sham group has no expression of active caspase-3. Expression of active caspase-3 in SAH + vehicle group increase obviously (*P < 0.01) and it decreased significantly in SAH + methazolamide group (^#^*P *< 0.05 *vs* SAH + vehicle group). N = 5 mice for each group.

We analyzed the expression of active caspase-3 in cortex and hippocampus of bleeding side. Our immunostaining results showed a low positive rate of active caspase-3 in the sham group, and the positive rate of active caspase-3 in the SAH + vehicle group were increased (*P *< 0.01) compared with sham group, while the positive rate of SAH + methazolamide group was lower than the SAH + vehicle group (*P *< 0.05). Figure 5a shows the representative images of immunostaining of active caspase-3 in cortex and hippocampus and Fig. 5b shows the positive rate of active caspase-3 in different groups in cortex and hippocampus of animals.Western blot quantification further confirms the immunostaining results by showing that the significantly increased expression of cleaved caspace-3 (17 kDa in Fig. 5c) compared with the ones in sham group (about 1.6 fold vs sham group), while the administration of methazolamide significantly inhibited the expression of active caspase-3 (about 1.2 fold *vs*. sham group) (*P* < 0.01, Fig. 5d).

### The neuroprotective effects of methazolamide in a cellular model of SAH

We wonder whether methazolamide can provides neuroprotection not only in mouse model of SAH *in vivo*, but also in cellular model of SAH *in vitro*. Since SAH refers to blood bleeding within the subarachnoid space, and hemoglobin-induced cytotoxicity in PCNs^18^, we firstly exposed PCNs to blood.

**Figure 6:**
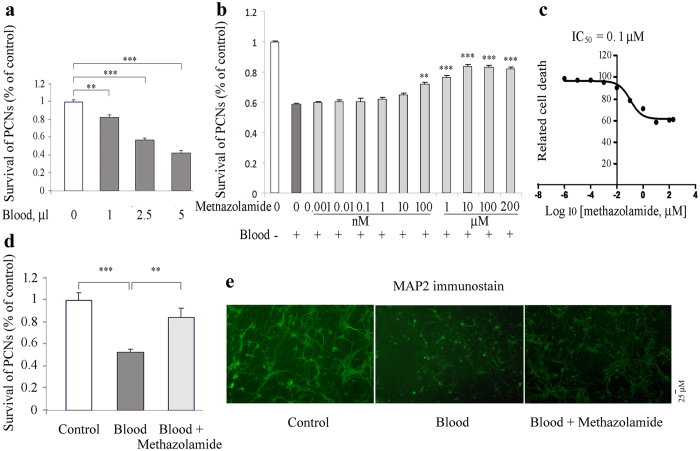
Methazolamide inhibits blood-induced PCNs cell death. Blood samples are collected using retroorbital vein method in mice. Cell death of PCNs is induced by 48-hour exposure to blood: 0 (100 μl culture medium), 1 (99 μl medium), 2.5 (97.5 μl medium) or 5 μl blood (95 μl medium) (**a**) or 2.5 μl blood are added to 97.5 ml medium with or without a series of concentrations of methazolamide (**b,c**) or 10 μM methazolamide (**d,e**). PCNs are then fixed with 4% paraformadehyde and immunostained with MAP2 antibody. Cell death was evaluated by the MAP2 immunostaining and counting (**a–d**). The number of survived PCNs per well are summed from the fields counted and expressed as a percent of those present in control wells not exposed to blood. Data from 3 independent experiments are graphed, and statistically significant differences are indicated with **P < 0.01, ***P < 0.001. The extent of cell death is always normalized relative to what is measured in the absence of both death stimulus and methazolamide (white bar). The dark grey bar corresponds to the extent of cell death in response to the blood without the test drug. Light gray bars correspond to the extent of cell death with blood and methazolamide. The relative cell death results are displayed graphically. The resulting curves (plotted semilogarithmically) define the IC50 of methazolamide (**c**). (**e**) PCNs are grown in 96-well plates. Representative survival counting and images of immunohistochemistry staining of MAP2 are shown (**d, e**). Scale bar = 25 mm.

**Figure 7:**
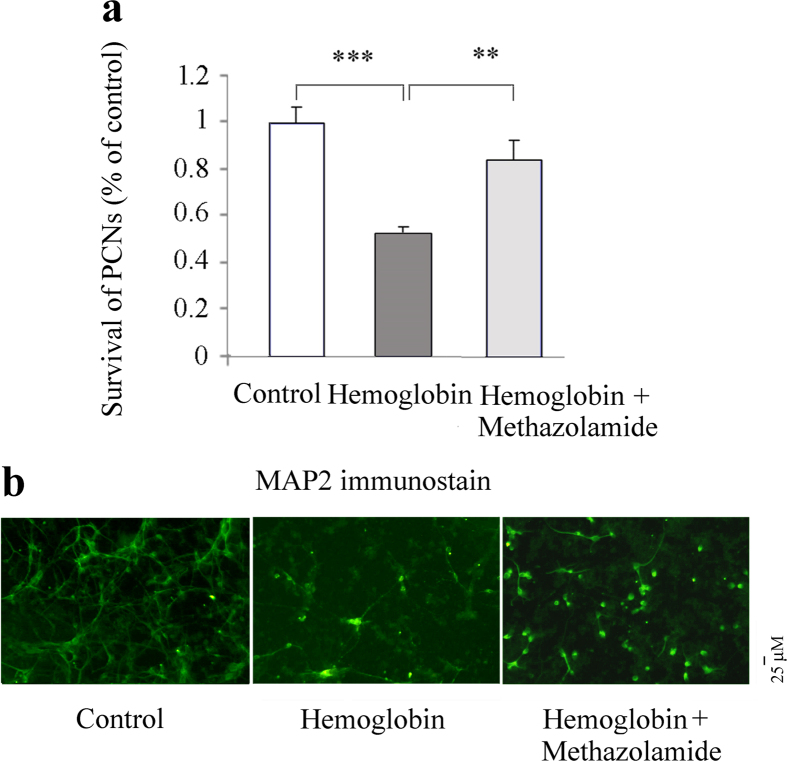
Methazolamide inhibits hemoglobin-induced PCNs cell death. Cell death of PCNs is induced by 48-hour exposure to 5 μM hemoglobin with or without methazolamide. PCNs are fixed and immunostained with MAP2 antibody. The number of survived PCNs per well are summed from the fields counted and expressed as a percent of those present in control wells that not exposed to hemoglobin. Cell death was evaluated by the MAP2 immunostaining and counting (**a**) (as in Fig. 6d,e). Methazolamide (10 μM) is pre-treated 2 h in right panel. Representative images of immunohistochemistry staining of MAP2 are shown (**b**). Scale bar = 25 μm. Data from at least 3 independent experiments.

MAP-2 is a neuron-specific cytoskeletal protein that is used as a marker of neuronal phenotype. Using MAP2 immunostaining, PCNs were observed and the number of PCNs in different treatments was summed from the fields counted under fluorescent microscopy and expressed as a percent of those present in control wells that not exposed to blood (Fig. 6a). The counting results showed that the exposure of PCNs to blood induces a dose-dependent cytotoxicity (Fig. 6a). 1, 2.5, or 5 μl blood all cause PCN cell death. To provide evidence of methazolamide protection against neuronal cell death, we choose 2.5 μl blood to measure the extent of cell death as a function of methazolamide (a series of concentrations from 0.0001 nM to 200 μM). The resulting curve (plotted semilogarithmically) of survival PCNs data (Fig. 6b) defines the IC_50_ of methazolamide as 0.1 μM and 25.4% as maximum protection (Fig. 6c). Hemoglobin, the important blood component, is neurotoxic, and has been reported to induce cytotoxicity in rat cerebral cortical neurons^19^. We further tested whether hemoglobin also induces mouse PCNs cell death. Indeed, our survival data using MAP2 immunostaining showed that hemoglobin insult causes loss of neuronal cells, while the administration of methazolamide significantly inhibits the neuronal loss (Fig. 7a,b).

MAP-2, a protein normally found in the somatodendritic compartment of neurons, may play a role in determining and stabilizing dendritic shape/length and is accordingly observed only within the dendritic arbor^20^.Morphometric analyses of MAP2-stained PCNs as previously described^21^ showed that blood exposure or hemoglobin not only causes loss of neuronal cells compare with the control PCNs (Figs 6d and 7a), but also causes the morphological changes including the loss of axo-dendritic arborization of PCNs and shortening of dendritic length of PCNs (Figs 6e and 7b, middle panels) compared with the regular axo-dendritic arborization and dendritic length of control PCNs (Figs 6e and 7b, left panels). Interestingly, the administration of methazolamide recovered not only the blood- or hemoglobin-induced loss of number of PCNs, but also partially restores normal neuronal morphology of PCNs including the regular axo-dendritic arborization and increased dendritic length of PCNs (Figs 6e and 7b, right panels). Our results thus demonstrate that neuroprotective effect of metazolamide in the inhibition of blood- or hemoglobin-induced loss of number of PCNs and recovery of normal neuronal morphology of PCNs.

### Methazolamide administration reduces oxidative stress *in vitro*

Oxidative stress plays a significant role in the development of acute brain injury and cerebral vasospasm following SAH. There are several sources for the excessive generation of free radicals following SAH, including extracellular hemoglobin, disrupted mitochondrial respiration, and the upregulation of free radical producing enzymes^22^. It has been reported that Reactive Oxygen Species (ROS) describes a number of reactive molecules and free radicals derived from molecular oxygen. SAH can induce ROS production in animals^23^, while hemoglobin caused an elevated intracellular ROS levels in primary neurons^19^.

**Figure 8:**
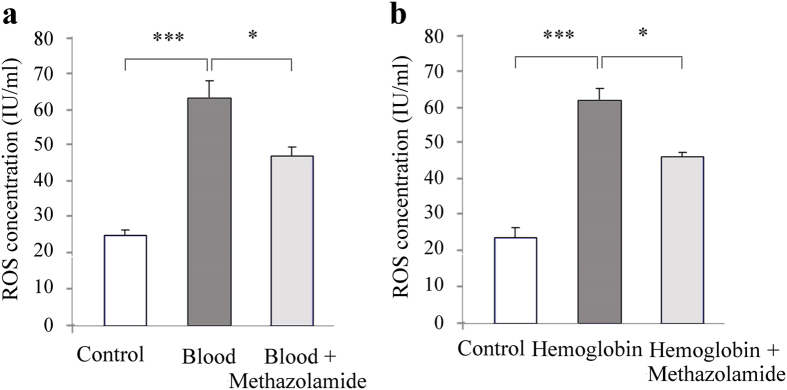
Mathazolamide effectively inhibits ROS production in blood- or hemoglobin- induced primary cortical neurons. PCNs grown in 96-well plates and exposed to blood (**a**, 2.5 μl blood are added to 97.5 μl medium with or without methazolamide) or hemoglobin (**b**, 5 μΜ hemoglobin, either with or without methazolamide) for 18 h. PCNs were trypsinized and centrifuged for 10 min at 3000 rpm. The supernatants were submitted for interacellular ROS (IU/ml) measurement. The quantitative analyses of interacellular ROS levels were shown. Data from 3 independent experiments.

We next tested whether methazolamide can offer neuroprotection through its effect on ROS using mouse ROS ELISA kit. Our results are consistent with previous findings that hemoglobin or blood significantly elevated intracellular ROS levels in cultured PCNs. Interestingly, we demonstrate that the administration of methazolamide could effectively inhibit ROS production induced by blood (Fig. 8a) or hemoglobin (Fig. 8b). As our knowledge, this is first report that methazolamide, as an antioxidant agent does, effectively inhibit ROS production induced by hemoglobin insult.

## Discussion

Methazolamide is a carbonic anhydrase inhibitor, which can penetrate the BBB due to its small molecule. It has been confirmed the effect of neuroprotection in recent years by us and other investigators^24^,^25^. However, there is no literature reported whether methazolamide has a protective effect on brain injury after SAH. Therefore our study, as this first report, used the vascular puncture SAH mice model *in vivo* to investigate the effects of methazolamide on nerve dysfunction and neuronal apoptosis of SAH (Figs 1, 2, 3, 4, 5), the finding is further supported by our data demonstrating methazolamide offers neuroprotection in blood- or hemoglobin-treated primary cortical neurons *in vitro* (Figs 6, 7, 8).

Here we found there was no significantly difference of amount bleeding in each group in this experiment, as well as no significantly difference of fluctuation in cererbral flow during SAH, which means no bias in our conclusion about the effect of methazolamide. Previous studies has focused on the delayed vasospasm and complications of SAH, however, up to date, still no effective treatment to prevent and reduce the brain damage in patients with SAH^15^. Thus it led to the current high mortality and morbidity post-SAH. Finding effective measures to mitigate brain damage of SAH is a challenging target for neurosurgeons.

We used neurological score to evaluate the nerve damage and found that the recovery process of the methazolamide group recovered quickly than the untreated group (Fig. 2). These suggest that methazolamide improve neurological score after SAH in mice. Generally, SAH leads to a sharp increase in intracranial pressure, then reduces cerebral blood flow. These initial changes are of physiological regulatory mechanism to reduce the intracranial hemorrhage^26^,^27^. With the disorders of the regulatory mechanism, brain perfusion is obviously insufficient, leading to cerebral ischemia, cerebral edema, eventually leading to nerve damage. Brain edema is an important symbol of secondary brain injury and pathophysiological processes after SAH, which will aggravate brain ischemia and hypoxia^28^. Therefore, improving brain edema can effectively reduce brain damage. As brain water content reflected the severity of brain edema after SAH and methazolamide decreased brain water content, it suggested methazolamide can reduce brain edema in SAH mice (Fig. 2), and may be one reason for the early improvement of neurological damage.

Aquaporins (AQPs) are water-permeable channels that provide the main route for water movement across the membrane in many cells. AQPs were upregulated in the ischemic area and found in astrocytes at the beginning of ischemic stroke^29^ More details, AQP1, AQP4, AQP5 and AQP9 were found to be expressed in the brain. In this study, we demonstrate that methazolamide suppressed brain edema. Report demonstrates that methazolamide’s analog acetazolamide, another potent carbolic anhydrase inhibitor, reversibly inhibits water conduction by aquaporin-4, but not through AQP1, while methazolamide shows no significant effect on water conduction by AQP4 or AQP1 in a liposome system in where purified recombinant rat AQP4 and human AQP1 were reconstituted^30^. The molecular mechanism for the methazolamide suppressed brain edema may be associated with AQPs in our SAH model. However, whether methazolamide inhibits brain edema by AQPs and which AQP may be involved remain to be elucidated.

Numerous studies confirm that SAH can cause cognitive, learning and memory dysfunction, thereby affecting prognosis^31^. The animal experiments showed that after SAH ipsilateral hippocampal neuronal damage is obvious^32^. Because the hippocampus is located within the temporal lobe brain, forms part of the limbic system, plays on the role of memory and spatial orientation. Hippocampal damage, manifested symptoms of memory loss and disorientation. Therefore, it is believed that the damage of hippocampal neurons may be responsible for the biological basis of learning and memory after SAH cognitive dysfunction. Open-field test is used to evaluate cognitive function in experiment that reflects the independent behavior of animals in a new environment, such as exploratory behavior and excitability. In present study, open-field test indicated that methazolamide could not only reduce the early neurological damage in mice of SAH, but also effectively improved the medium-term cognitive function (Fig. 2).

The phase of brain injury after SAH can be divided into early brain injury (EBI) and delayed ischemic brain injury with 72 h after SAH as a dividing line. Broderick *et al.* put forward the early brain injury after SAH in 1994^33^. Now it is considered as pathophysiological processes that lead to the brain endothelial cells and neuronal necrosis and apoptosis, brain edema, BBB changes in acute cerebral vasospasm and microvascular dysfunction, due to the increased intracranial pressure and cerebral blood flow reduction^34^. Therefore, reducing the EBI is very important for the treatment and improve prognosis of SAH. In our experiments, methazolamide could improve neurological function, decrease cerebral edema early after SAH, suggesting methazolamide alleviate the early brain injury after SAH by some mechanisms.

Current research on the mechanism of neurological damage after SAH is less, however, there is still evidence of apoptotic neurons involved in neuronal injury after SAH^34^. Cahill *et al.*^35^ reported the use of p53 inhibitors could reduce the cortical and hippocampal neuronal apoptosis and improve the prognosis of animals. Gao *et al.*^36^ found that caspase inhibitors could reduce neuronal apoptosis and basal caspase protein levels of cortex and hippocampus in rats after SAH, meanwhile improving neurobehavioral function and cerebral edema. These results suggest that apoptosis plays an important role in neuronal injury after SAH, and the reduction of neuronal apoptosis after SAH can be one of the important strategies for neuroprotection.

However, Westermaier T *et al.*^37^ reported that the occurrence of acute cerebrovascular vasospasm (CVS) was related with the amount of bleeding, the cerebral blood flow reduction and the persistence of a high concentration of glutamate outside cells after SAH, and it may played an important role in neurological injury. However, we found the average diameter of the middle cerebral artery on bleeding side was significantly narrowed 24 h after SAH compared with the sham group, showing a significant cause of CVS 24 h after SAH. According to our results, there was no significant change on the average diameter of blood vessels between SAH + methazolamide group and SAH + vehicle group, suggesting that methazolamide may improve the neurological function without the mechanism of reducing vasospasm in SAH mice (Fig. 1).

In order to find the possible mechanisms of the protective effect of methazolamide in SAH, we therefore focused on neural apoptosis. FJB and TUNEL staining were used in our experiment, which had a high affinity for degeneration neurons. We discovered that apoptosis of neurons of cortical and hippocampus tissue was significantly reduced on bleeding side in the mice of methazolamide treatment after SAH, suggesting that methazolamide can reduce SAH-induced neuronal apoptosis (Figs 3 and 4). This provided evidence for the possible mechanisms of protection of methazolamide in SAH mice.

Neuronal apoptosis is a complex cascade involving many proteins processes, multiple apoptotic pathways have known to play a role in apoptosis of neurons, such as the death receptor pathway, caspase-dependent pathway and caspase-independent pathway^38^,^39^,^40^. Wang *et al.*^7^ found methazolamide could act on the cortical cells to inhibit the release of cytochrome C, thereby further reduced the activation of caspase-1 and caspase-3 to inhibit apoptosis.

Caspase is closely related to the apoptosis of eukaryotic animal. It has been found a total of 14 family members and can be divided into two categories: one involved in the activation of other caspase family members, including Caspase-1, 2, 4, 5, 8, 9 and 10; the other is mainly mediated the execution phase of apoptosis, including Caspase-3, 6, 7, 14^41^. Caspase-3 is the central link in apoptotic cascade, a key enzyme and the executor of apoptosis^42^. Apoptosis is triggered by the integrity loss of mitochondrial outer membrane, release of apoptotic proteins such as cytochrome C, then activate caspase-3 to lead apoptosis^43^. In our experiment, the result showed that methazolamide could significantly reduce the content of activated caspase-3 of cortical and hippocampal cell on bleeding side in SAH mice (Fig. 5), indicating that methazolamide reduced the expression of active caspase-3 protein and activation factor-related apoptosis mainly by inhibiting caspase-dependent apoptosis pathway, thereby inhibited apoptosis of neurons to achieve the early improvement of neurological function and brain edema, medium-term improvement of cognitive function in SAH mice.

Primary cortical neurons *in vitro* model of SAH offer advantages over *in vivo* models. First, it makes possible to study the role of isolated neuronal cells as one pure particular type in an environment that simulates the SAH although neuronal cell death has been demonstrated *in vivo* SAH model. Second aspect, investigating mechanisms of protection of methazolamide is also facilitated. In present study, *in vitro* primary cultures have been used to provide important clues to dissect the mechanisms of SAH and potential actions of methazolamide. More details, using a simplified *in vitro* model of whole blood and hemoglobin neurotoxicity, we detect the neuroprotection of methazolamide against blood and hemoglobin-induced primary neuronal cell death (Figs 6 and 7) and demonstrate that the neuroprotection is through the inhibition of elevation of intracellular ROS levels (Fig. 8).

Studies reported that oxidative stress is involved in the pathogenesis of early brain injury and antioxidant can be one of the therapeutic candidates for early brain injury after experimental SAH^44^,^45^. Some antioxidant therapies have also demonstrated improved outcomes in clinical trials. These studies have laid a foundation for the use of antioxidants in the treatment of aneurismal SAH^46^. ROS plays an important role in the pathogenesis of cerebral vasospasm after SAH. Oxygen free radical scavengers can be mitigated oxyhemoglobin due to cerebral vasospasm. Our results indicated that intracellular levels of ROS significantly increased in PCNs after blood or hemoglobin insults. In this study, we found that methazolamide alleviated blood or hemoglobin -induced oxidative stress by at least partly eliminating intracellular levels of ROS (Fig. 8).

Besides oxidative stress, the inflammatory responses play a vital role and have been demonstrated in SAH pathogenesis including cellular responses, expression of adhesion molecules, production of intracellular inflammatory mediators, and accumulation of endothelial platelet aggregates. Inflammation persists through hemorrhage degradation in the subarachnoid space. Inflammatory aspects have been reported to lead to brain edema and cell death an inflammation is a particularly important therapeutic target for designing therapeutic strategies against secondary injuries after SAH^47^. In this study, our results showing that SAH causes brain edema (Fig. 2) and programmed cell death (Figs 4 and 5) support the possible involvement of inflammation in intravascular puncture mouse model of SAH. Furthermore, our results demonstrate that methazolamide improves neurological function and cognitive function (e.g., reducing brain water content in Fig. 2, offers anti-apoptosis effect (e.g., decreasing TUNEL stain in cortex and hippocampus in Fig. 4 and inhibiting caspase-3 activation in Fig. 5), they may imply that methazolamide improves neurological behavior by inhibition of inflammation in SAH mice.

In conclusion, this study showed that the treatment with methazolamide after SAH could inhibit brain edema, improve nerve function and cognitive function, reduce brain injury after SAH, while methazolamide offers neuroprotective effects and alleviated blood- or hemoglobin-induced neuron loss in cellular model of SAH. Our findings about the neuroprotection of methazolamide in cellular system and mouse animal model of SAH are thus remarkably consistent. Methazolamide could reduce the degeneration and apoptosis of cortex and hippocampal neurons on bleeding side, and this may be one of the mechanisms for the neuroprotective effect, which may be related to the activation of expression of active caspase-3. In addition, methazolamide inhibits blood- or hemoglobin-induced oxidative stress through at least partly eliminating intracellular levels of ROS. However, the specific protective mechanisms of methazolamide including the possible inhibition of inflammation need further research.

## Additional Information

**How to cite this article**: Li, M. *et al.* Methazolamide improves neurological behavior by inhibition of neuron apoptosis in subarachnoid hemorrhage mice. *Sci. Rep.* **6**, 35055; doi: 10.1038/srep35055 (2016).

## Supplementary Material

Supplementary Information
