# Supplementary material for: Methazolamide improves neurological behavior by inhibition of neuron apoptosis in subarachnoid hemorrhage mice
Source: Sci Rep. 2016 Oct 12;6:35055. doi: 10.1038/srep35055 (PMC5059745; doi:10.1038/srep35055)
Supplement: Supplementary Information [file srep35055-s1.pdf]

Methazolamide improves neurological behavior by inhibition of neuron apoptosis in subarachnoid hemorrhage mice

Mingchang Li, Wei Wang, Haojian Mai, Xinmu Zhang, Jian Wang, Yufeng Gao, Yuefei Wang, Gang Deng, Ling Gao, Shuanhu Zhou, Qianxue Chen, Xin Wang

Supplementary Information

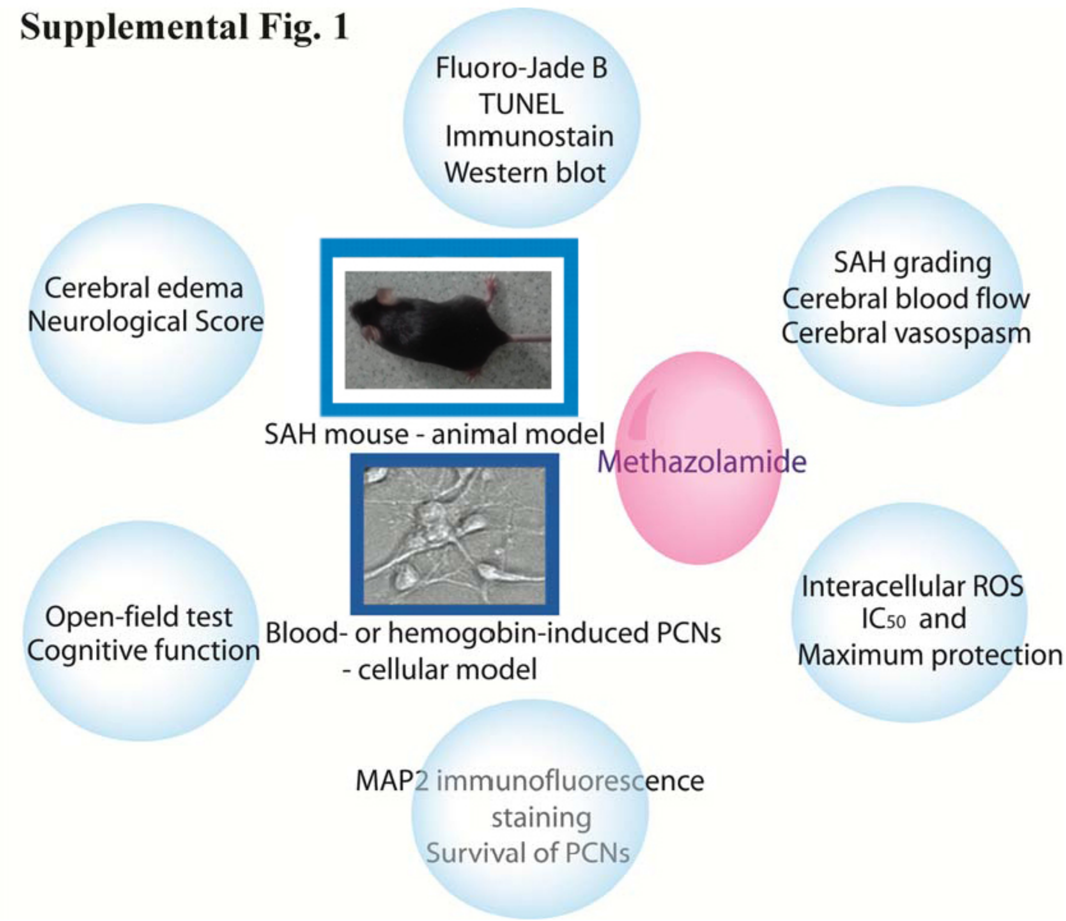

Supplemental Fig. 1 Scheme of neuronprotection of methazolamide. The protocols to investigate the neuroprotection of methazolamide in mouse model of SAH *in vivo* and in blood- or hemogobin-induced PCNs, a cellular model of SAH *in vitro* is schematized.
